# The study of screw placement parameters for Ogawa type I acromial fractures by 3D simulation

**DOI:** 10.1186/s13018-021-02416-3

**Published:** 2021-04-14

**Authors:** Wei Zhang, Zhongye Sun, Weiyan Li, Jun Yan, Liren Han, Shizhang Han, Xiaofei Yang, Bei Zhao

**Affiliations:** 1 Department of Orthopaedics, Liaocheng People’s Hospital, Dongchang West Road, Liaocheng, 252000 Shandong China; 2 Department of Orthopaedics, Gaotang People’s Hospital, Liaocheng, Shandong China

**Keywords:** Acromion, Screw fixation, Axial perspective, 3D technology

## Abstract

**Background:**

Acromial fractures are rare and there is no consensus on fixation, but an increasing number of studies have reported using two screws to fix Ogawa type I acromial fractures. The objective of this study was to obtain the ideal length, diameter, insertion point, and angle of the screw using a novel 3D simulation.

**Methods:**

The scapular CT data of 100 individuals were obtained to reconstruct 3D models. The transparency of the 3D model was then downgraded along the axial perspective (the view perpendicular to the cross section of the acromion axis) to find the maximum translucent area. Two virtual screws were placed at the anterior edge of the acromion until they penetrated the posterior cortical bon. The largest diameters and lengths of the screw were measured, and the direction and insertion point of the screw were observed.

**Results:**

The mean maximum lengths of the medial and lateral screws were 43.33 ± 6.17 mm and 39.23 ± 6.01 mm, respectively. The mean maximum diameters of the medial and lateral screws were 4.71 ± 1.23 mm and 4.97 ± 1.07 mm, respectively. Differences in screw length, diameter, and insertion point between males and females were found. The differences in screw angle between sexes were not statistically significant.

**Conclusions:**

Based on a 3D model test, we recommend the size, entry points, and angles of screws for Ogawa type I acromial fractures, providing valuable guidance for clinical work. More accurate screw parameters can be obtained preoperatively by establishing an individualized 3D model.

## Introduction

Acromial fractures are rare injuries, accounting for approximately 8–16% of scapular fractures [1, 2]. The injury mechanism of fracture is mainly direct shoulder violence, indirect humeral head impingement, or complications following reverse total shoulder arthroplasty. Due to the weight of the upper limb and the pull of the deltoid muscle, the acromial bone block will shift, which leads to narrowing of the subacromial space, and the rotator cuff tears, resulting in shoulder pain and limited movement.

In 1997, Ogawa proposed a practical classification based on the location of the acromial fracture line and anatomical structure [3]. He classified acromial fractures as follows: type I fractures consist of those of the anatomic acromion and extremely lateral scapular spine. Type II fractures consist of those located in the more medial spine and descending to the spinoglenoid notch. This classification is recognized and used to guide treatment.

Unfortunately, there is no consensus on the treatment and fixation methods for acromial fractures. The usual fixation methods include Kirschner wires, tension bands, and anatomical locking plates [3–5]. However, in type I fractures, conventional plate fixation is not recommended because of the very thin and small nature of the osseous anatomy. Fixation with Kirschner wire cannot pressurize the fracture end and is prone to early fixation failure [6, 7].

At present, in the treatment of distal acromial fractures, the use of two cannulated screws instead of Kirschner wire fixation is considered an effective method that has a high postoperative fracture healing rate and no complications [8–11]. Peckett et al. reported on symptomatic acromial fracture fixation with two 3.5 mm screws in 17 patients, and the postoperative healing rate was 94% [9]. Garnon et al. demonstrated the good technical feasibility of percutaneous image-guided screw fixation for the treatment of pathological distal acromial fractures [11]. In previous studies, only screw diameters were reported, and the differences were large. No guidance was given regarding the length, insertion point, or ideal angle for the two screws.

3D simulation technology has been widely used in the field of orthopedic surgery to help surgeons understand anatomical structures (nerves, vessels) and anatomical parameters (length, angles, anatomical axis) [12, 13]. This technology has also been widely used to guide the treatment of bone tumors [14] and thermal necrosis [15]. To date, there have been many reports on the use of this technique to guide screw fixation of different parts of fractures. However, there has been no report on the use of 3D simulation to guide screw fixation for acromial fractures. The objective of this study was to obtain the implantation point, optimal axial angle, diameters, and lengths of the two screws by using 3D simulation.

## Materials and methods

**Fig. 1:**
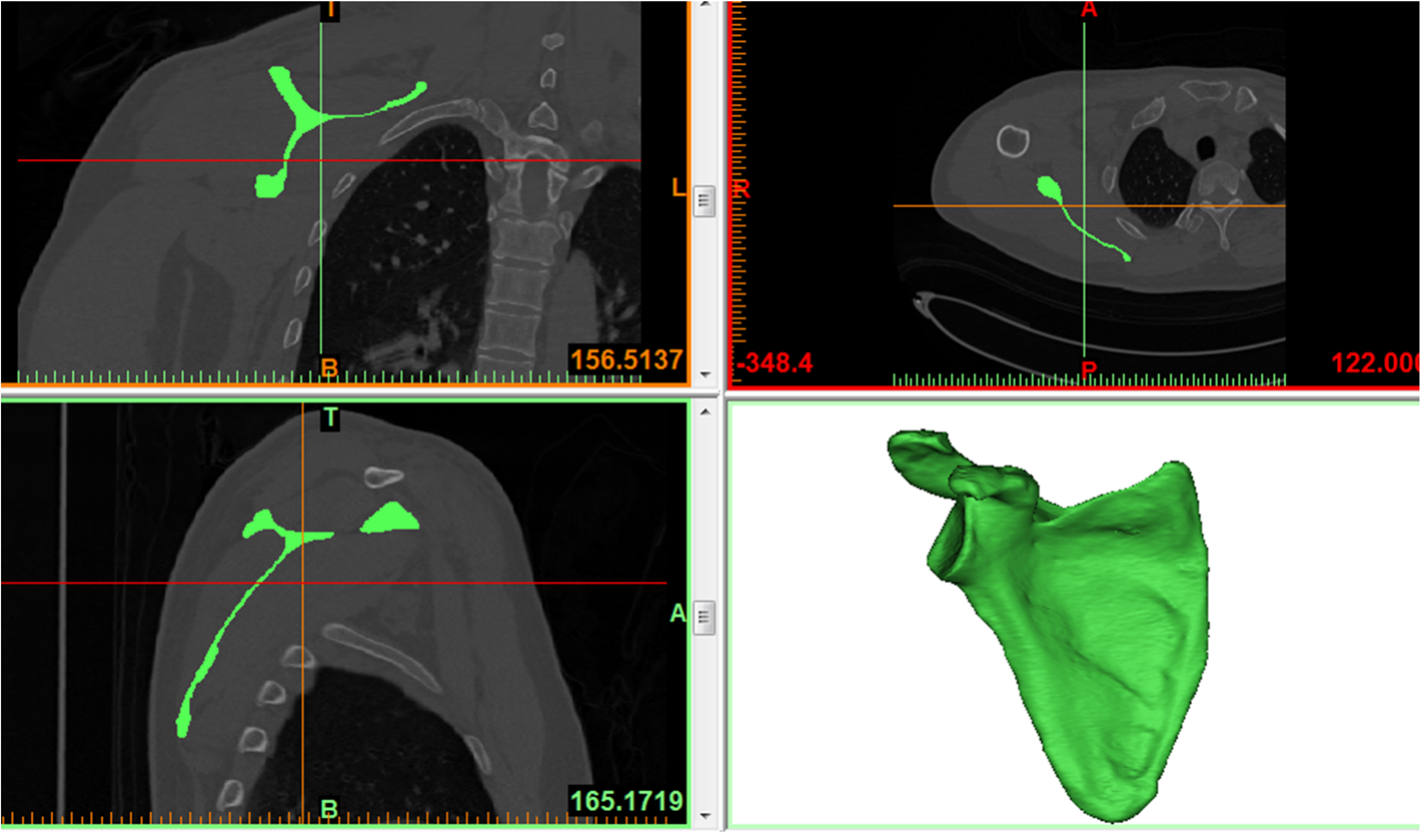
The 3D models of the scapula was obtained through image segmentation and region growth operations of the software

One hundred Chinese individuals without fractures or lesions of the right scapula were enrolled between January 2019 and November 2020 in this study. There were 50 males and 50 females. The mean age of the patients was 54.21 ± 15.42 (range 20–85). All patients received 64-slice spiral CT continuous slice scans in our hospital, and the original data were obtained in DICOM format. All of the original data were imported into Mimics software one by one. The 3D model of the scapula was obtained through image segmentation and regional growth operations of the software (Fig. 1).

**Fig. 2:**
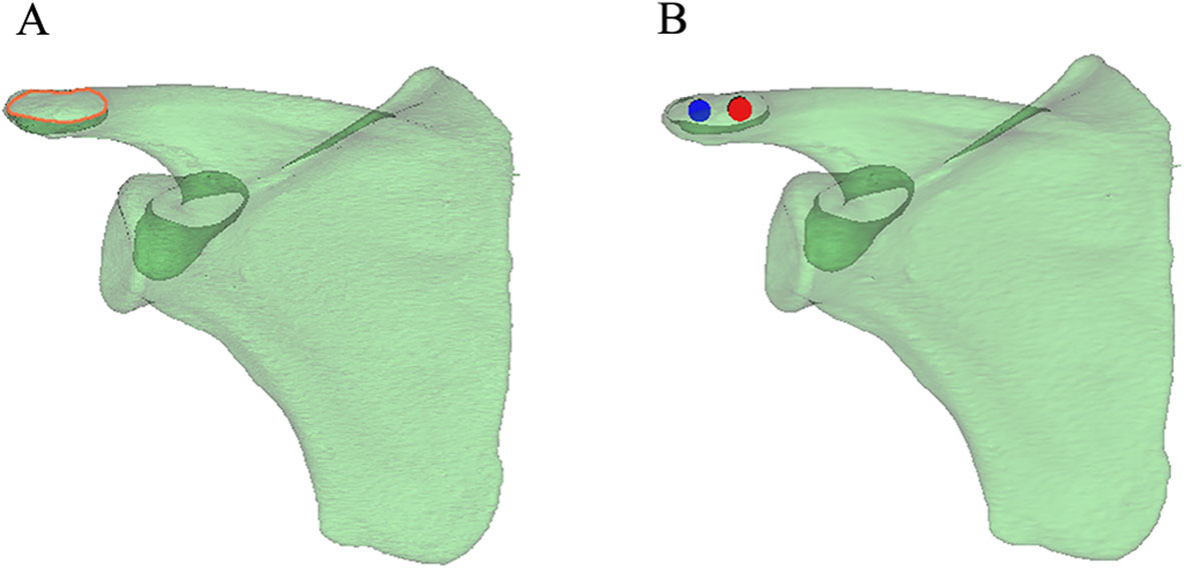
Find the largest screw path. **a** The largest translucent area resembles an oval shape. **b** Two computer-aided design screws were placed evenly in the translucent area. Then, the diameters were increased progressively until they reached the borderline of the area

**Fig. 3:**
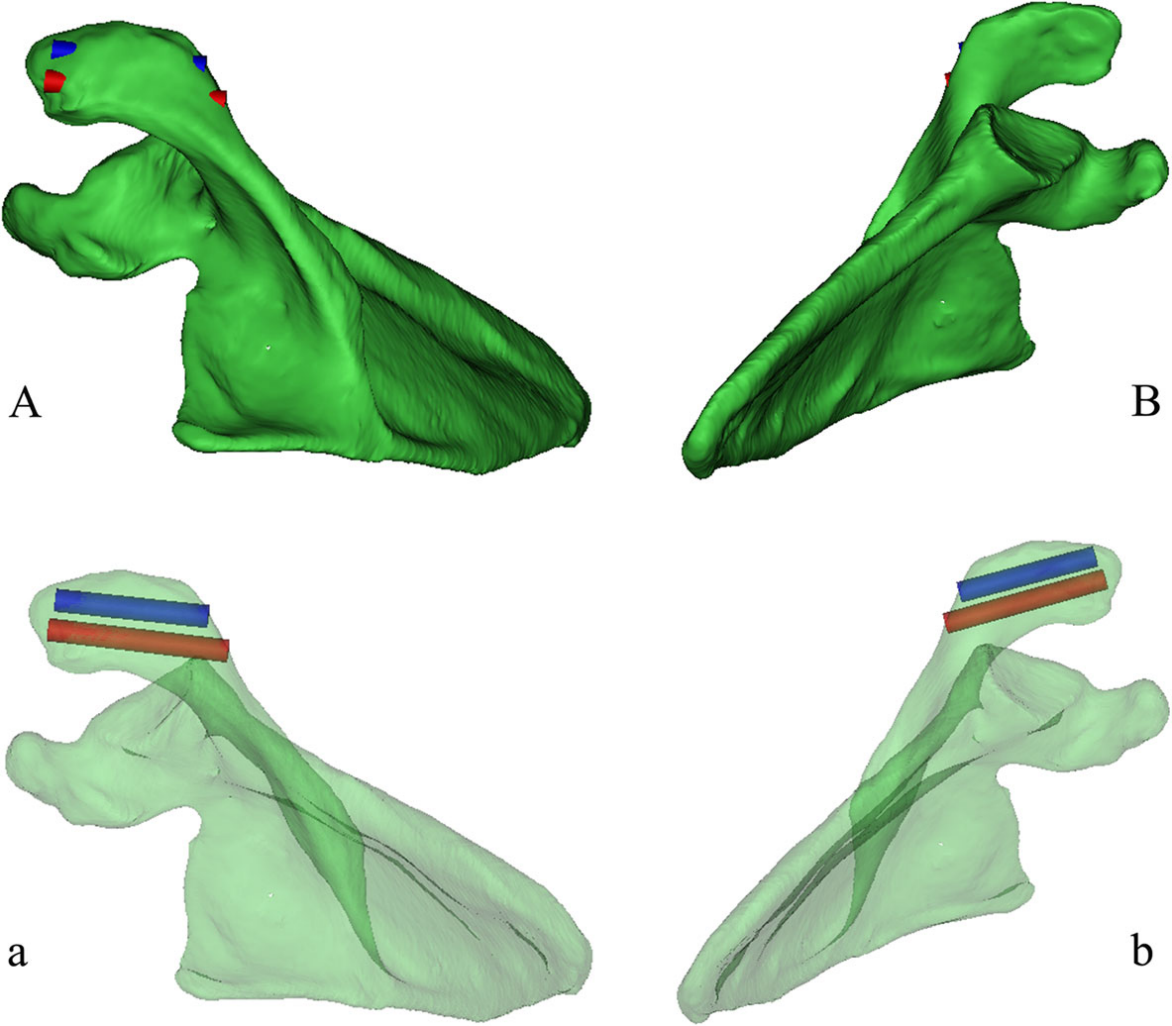
Observe the largest length and position of the screws. **A**, **B** Observed from the above and below of the opaque 3D model, respectively. The screws had the largest lengths and diameters just penetrating the cortical bone. **a**, **b** The screws position were observed from the above and below of the translucent 3D model.

**Fig. 4:**
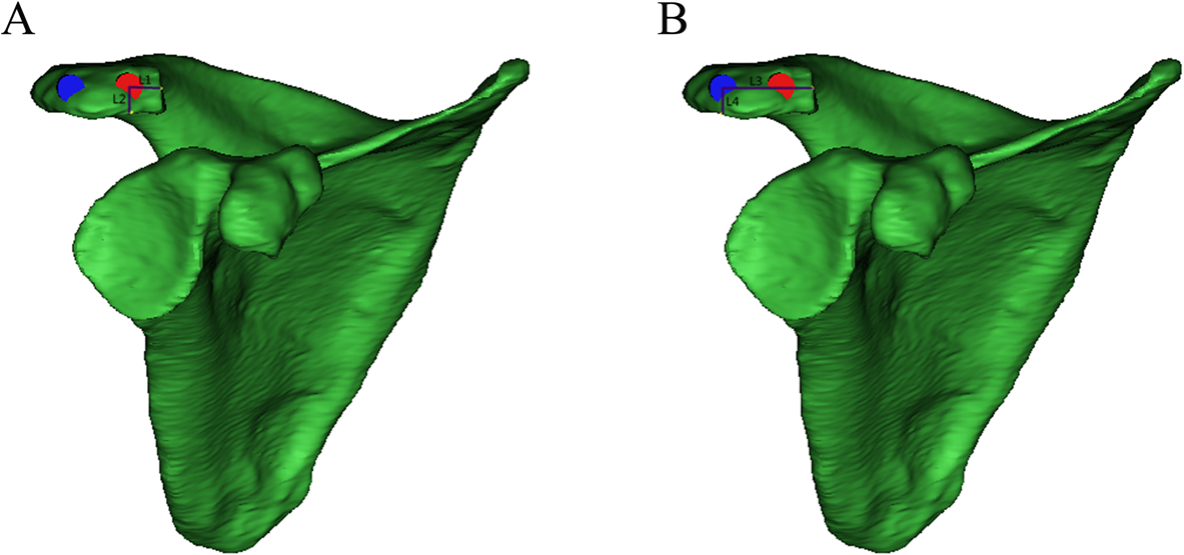
The measurement of distance L1, L2, L3, and L4. **a** The distances from the medial screw entry point to the acromioclavicular articular surface and the leading edge of the acromial were marked as L1 and L2, respectively. **b** The distances from the lateral screw entry point to the acromioclavicular articular surface and the leading edge of the acromial were marked as L3 and L4, respectively

**Fig 5:**
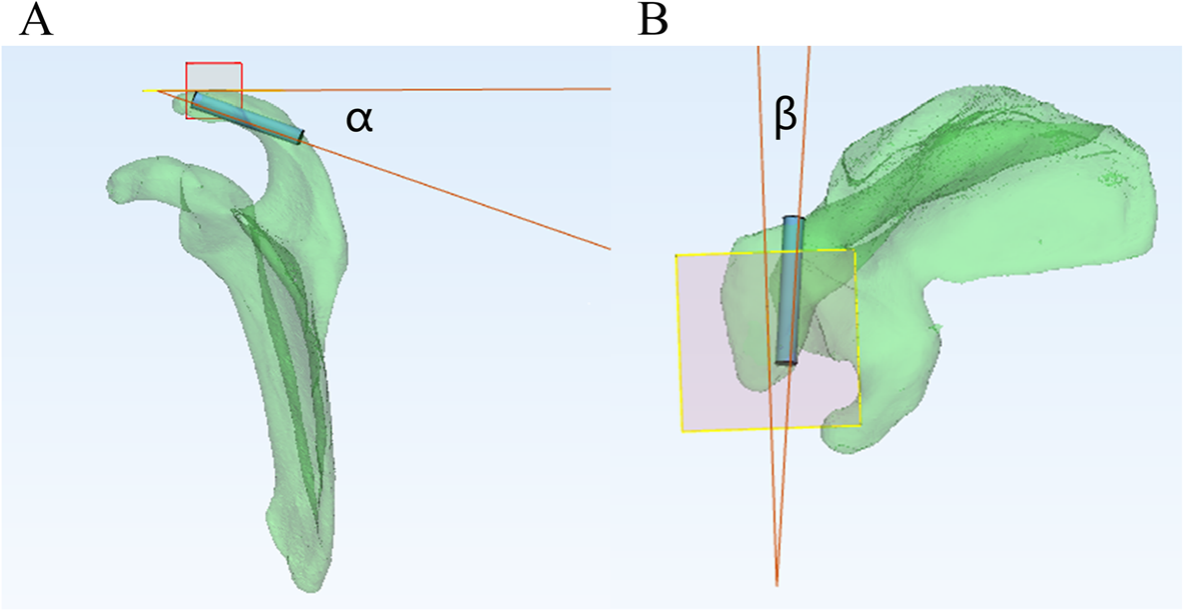
The measurement of angle α and β. **a** The downdip angle between the screw and plane A was measured and recorded as angle α. **b** The inclination angle between the screw and plane B was measured and recorded as angle β

To determine the effective screw passage, we reduced the transparency of the 3D model and rotated the 3D model to an axial view, which was parallel to the cross section of the distal acromion. The outline of a translucent area resembling an oval shape was clearly shown (Fig. 2a). We observed and adjusted the position of the model to maximize the translucent area and divided it evenly into two parts. Two computer-aided design screws were placed perpendicular to the translucent zone and gradually increased in diameter, defined as the maximum diameter when the screws did not penetrate the boundary of the zone (Fig. 2b). The screw length was then adjusted until it had just penetrated the posterior bone cortex, and the value was recorded (Fig. 3a,b). The anatomical markers of the acromioclavicular articular surface and the distal anterior edge of the acromion are easily accessible and recognized. To determine screw location, the distance from the insertion point to the acromioclavicular articular surface and the distal anterior edge of the acromion was marked. The L1 and L2 distances for the medial screw (MS) and the L3 and L4 distances for the lateral screw (LS) were recorded (Fig. 4a, b). The upper plane of the distal acromion was selected as the reference plane, which is called plane A. The downdip angle between the screw and plane A was measured and recorded as angle α (Fig. 5a). A plane perpendicular to plane A was defined as plane B. The inclination angle between the screw and plane B was measured and recorded as angle β (Fig. 5b).

The experimental data were analyzed by SPSS 25.0 statistical software. All continuous variables are presented as the mean and standard deviation. A *t* test was used to compare the data between males and females. Statistical significance was accepted at *P* < 0.05.

## Results

The reconstructed scapula model shows the safety zone of the acromial screw, as shown in Fig. 2.

Tables 1 and 2 the mean maximum lengths of the medial and lateral screws were 43.33 ± 6.17 mm and 39.23 ± 6.01 mm, respectively. The mean maximum diameters of the medial and lateral screws were 4.71 ± 1.23 mm and 4.97 ± 1.07 mm, respectively. The mean L1 distance was 7.25 ± 1.71 mm, the mean L2 was 6.38 ± 1.82 mm, the mean L3 was 17.04 ± 2.27 mm, and the mean L4 was 5.89 ± 1.63 mm. In the above data, the differences between males and females were statistically significant (*P* < 0.05).

**Table 1:** Differences in medial screw between males and females

Group	Length (mm)	Diameter (mm)	L1 (mm)	L2 (mm)
All	43.33 ± 6.17	4.71 ± 1.23	7.25 ± 1.71	6.38 ± 1.82
Male	50.79 ± 4.33	5.69 ± 0.81	7.33 ± 1.69	6.88 ± 1.89
Female	41.87 ± 4.19	3.73 ± 0.67	6.17 ± 1.73	5.87 ± 1.59
*t*	10.478	13.165	0.494	2.875
*P*	0.000	0.000	0.000	0.005

The differences between males and females were of statistical significance (*P* < 0.05)

**Table 2:** Differences in lateral screw between males and females

Group	Length (mm)	Diameter (mm)	L3 (mm)	L4 (mm)
All	39.23 ± 6.01	4.97 ± 1.07	17.04 ± 2.27	5.89 ± 1.63
Male	43.26 ± 4.64	5.81 ± 0.76	17.79 ± 2.26	6.48 ± 1.72
Female	35.20 ± 4.28	4.13 ± 0.56	16.29 ± 2.03	5.29 ± 1.28
*t*	9.033	12.478	3.497	3.930
*P*	0.000	0.000	0.001	0.000

The differences between males and females were of statistical significance (*P* < 0.05)

The mean *α* and *β* angles for males and females are recorded in Table 3. The mean *α* angles and *β* angles were 13.98 ± 5.03° and 6.53 ± 5.10°, respectively. The differences between sexes were not statistically significant (*P* > 0.05).

**Table 3:** Differences between males and females: angles *α* and *β*

Group	*α* (°)	*β* (°)
All	13.98 ± 5.03	6.53 ± 5.10
Male	13.53 ± 4.93	7.07 ± 4.88
Female	14.03 ± 5.14	6.99 ± 5.31
*t*	-0.941	1.061
*P*	0.375	0.291

The differences sexes were not statistical significance (*P* > 0.05)

## Discussion

The acromion is an important part of the superior shoulder suspensory complex. Acromial fractures are considered a special type of intra-articular fractures. If acromial fractures are not properly treated, they will inevitably affect the function of the shoulder joint. Complications associated with nonoperative treatment of displaced acromial fractures have been reported, including painful stiff shoulder, activity limitation, symptomatic nonunion, acromioclavicular joint separation, and subacromial impingement [1, 8, 12, 16–21]. Therefore, early surgical intervention is considered reasonable.

Acromial fractures are rarely reported in the literature, and there are no consistent treatment plans. Owage [3] recommends that patients with type I acromial fractures be treated with Kirschner wire or tension band fixation. However, there are reports of Kirschner wire loosening, fracture redisplacement, and needle tract infection after the use of this fixation method [6, 7, 22]. We also do not recommend the use of Kirschner wires because they do not provide adequate compression at the fracture site.

Screw fixation has been gradually recognized for its ability to provide adequate fracture compression and offer satisfactory fracture stability [7–11, 20]. Peckett et al. [9] recommended the use of double tension screws instead of Kirschner wires in 26 patients with acromial fractures. Kim et al. [8] reported fracture fixation with two cannulated screws in 27 patients without postoperative complications, such as screw displacement or local infection. Unfortunately, recommendations on the maximum diameters and lengths and the optimal entry points and appropriate angles of two screws for distal acromial fractures have not been reported.

The application of 3D computer models in the field of orthopedics is mature and reliable [23]. This method is used to conduct big data research and collect relevant data to provide help for surgeons to avoid the aggravation of fractures and screw loosening caused by repeated adjustment of screw direction and replacement of screws during operation.

In the past, screws of different diameters have been used to treat acromial fractures, including 3.0 mm, 3.5 mm, 4.0 mm, and even 5.0 mm [8–11]. According to the information in our study, the maximum diameters were 5.69 ± 0.81 mm (MS) and 5.81 ± 0.76 mm (LS) in males and 3.73 ± 0.67 mm (MS) and 4.13 ± 0.56 mm (LS) in females. This is consistent with previously reported screw sizes. We recommend the use of at least 3.5 mm screws in females and at least 5.0 mm screws in males. We also recorded the lengths of the two screws. The lengths of the screws were 50.79 ± 4.33 mm (MS) and 43.26 ± 4.64 mm (LS) in males and 41.87 ± 4.19 mm (MS) and 35.20 ± 4.28 mm (LS) in females. Due to individual and sex differences, we recommend preoperative evaluation and measurement of imaging data.

The shape of the acromion varies from person to person. Bigliani et al. divided patients into three types according to acromial morphology, in which the proportion of curved and hooked acromions was 81.6% [24]. This division not only increases the difficulty of screw implantation but also increases the chance of screw penetration into the subacromial space. Therefore, the insertion point and direction are two important indexes that affect the safe placement of the screw. In our study, we found that the distance from the entry point to the acromioclavicular articular surface and the distal anterior edge of the acromion was greater in males. This can be caused by a large shoulder blade in males. We recommend maximizing the inclination angle of the screw because the screw pointing to the base of the scapular spine provides stronger fixation.

We applied this model in the study of the acromions of 100 individuals, a sufficiently large sample size. As the axial perspective is quite similar to the X-ray projection, the two screw parameters we obtained can provide valuable guidance to surgeons. Given that the standard deviation of our results is relatively large, indicating that there are large differences between individuals, it is necessary to perform preoperative planning for each patient.

There are some limitations in our research. First, we studied the acromions of only Chinese people, and these data may not be applicable to people from other countries. Second, these software tools do not replace experimental testing; they provide a valuable and rapidly evolving option for evaluating implant designs at an early stage of the test [25], but further cadaver or clinical studies are needed to verify the accuracy of the technique.

## Conclusion

Through 3D model testing, we recommend the size, entry points, and angles of screws for Ogawa type I acromial fractures, providing valuable guidance for clinical work. More accurate screw parameters can be obtained preoperatively by establishing an individualized 3D model. In the near future, we hope to verify the strength and effectiveness of screws through biomechanical and clinical studies.

## Data Availability

The datasets generated and analyzed during the current study are available from the corresponding author on reasonable request.

## Abbreviations

3D: Three-dimensional
CT: Computed tomography
DICOM: Digital imaging and communication in medicine
Mimics: Materialise’s interactive medical image control system
SPSS: Statistical package for the social sciences
MS: The medial screw
LS: The lateral screw

## References

[1] Goss TP (1996). The scapula: coracoid, acromial, and avulsion fractures. Am J Orthop (Belle Mead NJ).

[2] Lantry JM, Roberts CS, Giannoudis PV (2008). Operative treatment of scapular fractures: a systematic review. Injury..

[3] Ogawa K, Naniwa T (1997). Fractures of the acromion and the lateral scapular spine. J Shoulder Elb Surg.

[4] Hertel R, Windisch W, Schuster A, Ballmer FT (1998). Transacromial approach to obtain fusion of unstable os acromiale. J Shoulder Elb Surg.

[5] Rupp S, Seil R, Kohn DM (1998). Surgical reconstruction of a stress fracture of the acromion after arthroscopic subacromial decompression in an elite tennis player. Arthroscopy..

[6] Bauer G, Fleischmann W, Dussler E (1995). Displaced scapular fractures: indication and long-term results of open reduction and internal fixation. Arch Orthop Trauma Surg.

[7] Konstantinidis GA, Smithers T, Hong TF (2020). Postoperative results of Ogawa type IIB meta-acromion fracture fixation with a 90° twisted reconstruction plate. Arch Orthop Trauma Surg.

[8] Kim DS, Yoon YS, Kang DH (2010). Comparison of early fixation and delayed reconstruction after displacement in previously nondisplaced acromion fractures. Orthopedics..

[9] Peckett WR, Gunther SB, Harper GD, Hughes JS, Sonnabend DH (2004). Internal fixation of symptomatic os acromiale: a series of twenty-six cases. J Shoulder Elb Surg.

[10] Cicekli O, Akar A, Topcu HN. Displaced acromion fracture: a rare injury, case report. J Surg Case Rep. 2017:313–6. 10.1016/j.ijscr.2017.08.051 PMC5602832 28898793

[11] Garnon J, Koch G, Ramamurthy N. Percutaneous CT and fluoroscopy-guided screw fixation of pathological fractures in the shoulder girdle: technical report of 3 cases. Intervent Radiol. 2016:1332–8. 10.1007/s00270-016-1333-2 27048488

[12] Preece D, Williams SB, Lam R, Weller R (2013). “Let’s Get Physical”: advantages of a physical model over 3D computer models and textbooks in learning imaging anatomy. Anat Sci Educ.

[13] Mediouni M, Volosnikov A (2015). The trends and challenges in orthopaedic simulation. J Orthop.

[14] Mediouni M, Schlatterer DR (2017). Orthopaedic tumors: what problems are we solving, and are universities and major medical centers doing enough?[J]. J Orthop.

[15] Mediouni M, Schlatterer DR, Khoury A, et al. Optimal parameters to avoid thermal necrosis during bone drilling: a finite element analysis [J]. J Orthop Res. 2017;35(11). 10.1002/jor.23542 28181707

[16] Kuhn JE, Blasier RB, Carpenter JE (1994). Fractures of the acromion process: a proposed classification system. J Orthop Trauma.

[17] Lim KE, Wang CR, Chin KC, Chen CJ, Tsai CC, Bullard MJ (1996). Concomitant fracture of the coracoid and acromion after direct shoulder trauma. J Orthop Trauma.

[18] Dounchis JS, Pedowitz RA, Garfin SR (1999). Symptomatic pseudarthrosis of the acromion: report of a case and review of the literature. J Orthop Trauma.

[19] McGahan JP, Rab GT (1980). Fracture of the acromion associated with an axillary nerve deficit: a case report and review of the literature. Clin Orthop Relat Res.

[20] Anavian J, Wijdicks CA, Schroder LK, Vang S, Cole PA (2009). Surgery for scapula process fractures: good outcome in 26 patients. Acta Orthop.

[21] Shindle MK, Wanich T, Pearle AD, Warren RF (2008). Atraumatic scapular fractures in the setting of chronic rotator cuff tear arthropathy: a report of two cases. J Shoulder Elb Surg.

[22] Hill B, Anavian J, Jacobson AR, Cole PA (2014). Surgical management of isolated acromion fractures technical tricks and clinical experience. J Orthop Trauma.

[23] Feng X, Zhang S, Luo Q, Fang J, Lin C, Leung F, Chen B (2016). Definition of a safe zone for antegrade lag screw fixation of fracture of posterior column of the acetabulum by 3D technology. Injury Int J Care Injured.

[24] Bigliani LU, Morrison DS, April EW (1986). The morphology of the acromion and its relationship to rotator cuff tears. Ortho Trans.

[25] Mediouni M, Kucklick T. Poncet, Sébastien, et al. An overview of thermal necrosis: present and future [J]. Curr Med Res Opin. 2019:1. 10.1080/03007995.2019.1603671 30943796

